# SRT1720 as an SIRT1 activator for alleviating paraquat-induced models of Parkinson's disease

**DOI:** 10.1016/j.redox.2022.102534

**Published:** 2022-11-11

**Authors:** Chih-Chang Chao, Chuen-Lin Huang, Jing-Jy Cheng, Chun-Tang Chiou, I-Jung Lee, Ying-Chen Yang, Ting-Huang Hsu, Chia-En Yei, Pei-Ying Lin, Jih-Jung Chen, Nai-Kuei Huang

**Affiliations:** a Institute of Neuroscience, National Chengchi University, Taipei, Taiwan; b Medical Research Center, Cardinal Tien Hospital, Hsintien, New Taipei City, Taiwan; c Graduate Institute of Physiology & Department of Physiology and Biophysics, National Defense Medical Center, Taipei, 114, Taiwan; d National Research Institute of Chinese Medicine, Ministry of Health and Welfare, Taipei, 112, Taiwan; e Institute of Biophotonics, National Yang-Ming University, Taipei, 112, Taiwan; f Herbal Medicine Department, Yokohama University of Pharmacy, Yokohama, Japan; g Department of Biotechnology and Animal Science, National Ilan University, Ilan, Taiwan; h Department of Medical Research, China Medical University Hospital, China Medical University, Taichung 404332, Taiwan; i Department of Pharmacy, School of Pharmaceutical Sciences, National Yang-Ming Chiao Tung University, Taipei 112304, Taiwan; j Ph.D. Program for Neural Regenerative Medicine, College of Medical Science and Technology, Taipei Medical University, Taipei, 11031, Taiwan; k Graduate Institute of Medical Sciences, College of Medicine, Taipei Medical University, Taipei, 11031, Taiwan

**Keywords:** Sirtuin1, SRT1720, Mitochondria, Paraquat, Parkinson's disease

## Abstract

Epidemiological studies have linked herbicides and Parkinson's disease (PD), with the strongest associations resulting from long exposure durations. Paraquat (PQ), an herbicide, induces PD-like syndromes and has widely been accepted as a PD mimetic. Currently, there is still no cure to prevent the progression of PD, and the search for effective therapeutic ways is urgent. Recently, the impairing activity of sirtuins (SIRTs), such as SIRT1, may correlate with PD etiology. However, the nonspecificity of SIRT1 agonists has made the protective mechanisms against PD unclear and hampered the therapeutic application of SIRT1. Thus, this study investigated the protective mechanism and therapeutic potential of SRT1720, a more specific agonist for SIRT1 synthesized by Sirtris, in alleviating the toxicity of PQ-induced cellular and animal models of PD. Here we show that SRT1720 alleviates PQ-induced toxicity in cell and animal models. Genetic silencing and pharmacological inhibition of SIRT1 attenuated SRT1720's protection against PQ-induced toxicity. Moreover, SRT1720 not only attenuated PQ-induced increased oxidative stress and mitochondrial free radical formations but also decreased mitochondrial membrane potential. Furthermore, SRT1720 reversed PQ-induced decreased PGC-1α levels and mitochondrial biogenesis. Although PQ and SRT1720 elevated NRF2 and antioxidative enzyme levels, only PQ decreased antioxidative enzyme activity but not SRT1720. *NRF2* and *PGC-1α* silencing attenuated SRT1720 protection against PQ-induced toxicity. SRT1720 targeted SIRT1 and activated downstream PGC-1α and NRF2 signalings to prevent PQ-induced toxicity involving oxidative stress and mitochondrial dysfunction. Thus, SRT1720 might have therapeutic potential in preventing PD.

## Highlights

• SIRT1 as the target of SRT1720 in mediating PQ-induced toxicities.
• SRT1720 prevents PQ-induced toxicities in cellular and animal models of PD.
• SRT1720 modulated PQ toxicity through PGC-1α pathway.
• SRT1720 modulated PQ toxicity through NRF2 pathway.

## Graphical abstract

**Figure:**
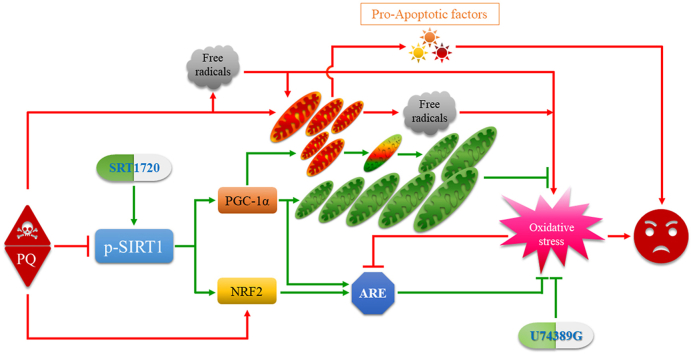

## Abbreviations

ANOVA: analysis of variance
BSA: bovine serum albumin; DAergic
AREs: antioxidant response elements; dopaminergic
DMEM: Dulbecco's modified Eagle's medium
ELISA: enzyme-linked immunosorbent assay
FBS: fetal bovine serum
FOXO: Forkhead-box transcription factor
GPX: glutathione peroxidase
GS: Goat serum
HO: heme oxygenase
ICC: immunocytochemistry
IHC: Immunohistochemistry
MPP^+^: 1-methyl-4-phenylpyridinium
MPTP: 1-methyl-4-phenyl-1,2,3,6-tetrahydropyridine;
NQO1: quinone dehydrogenase 1
NRF2: nuclear factor E2-related factor 2
6-OHDA: 6-hydroxydopamine;
PBS: phosphate-buffered saline;
PD: Parkinson's disease
PGC-1α: peroxisome proliferator-activated receptor gamma-assisted activating factor-1α
PQ: paraquat
PRDX: peroxiredoxin
PVDF: polyvinylidene difluoride;
ROS: reactive oxygen species
SD: standard deviation
SDS-PAGE: SDS-polyacrylamide gel electrophoresis
SIRTs: Sirtuins
sMaf: small musculoaponeurotic fibrosarcoma
SNpc: substantia nigra pars compacta
SOD1/2: superoxide dismutase 1/2
SRT: SRT1720
STACs: SIRT1 activating compounds
TH: tyrosine hydroxylase
TMRE: tetramethylrhodamine ethyl ester
TXN: thioredoxin
TXNRD: thioredoxin reductase
UPS: ubiquitin-proteasome system

## 1 Introduction

Parkinson's disease (PD) is the most prevalent movement disorder and represents the second most ordinary degenerative disease of the central nervous system, disturbing 1–2% of people over 65 years of age [85]. PD is categorized by the loss of dopaminergic (DAergic) neurons located in the substantia nigra pars compacta (SNpc) area and intracellular inclusions termed Lewy bodies that compose misfolded α-synuclein that progressively lead to motor and non-motor symptoms [52,64]. Epidemiologically, aging is recognized as a primary risk factor for developing PD [2]. Although the pathological mechanisms of the loss of DAergic neurons remain unclear, mitochondrial dysfunction, energy failure, oxidative stress, excitotoxicity, protein misfolding/aggregation, and autophagic flux impairment may involve the onset and progression of PD [55]. There is currently no remedy for PD, and only certain medicines, surgical treatments, and other therapies can relieve some of its symptoms [74]. Therefore, searching for and investigating novel mechanisms and therapeutic drugs for PD is necessary and urgent. Sirtuins (SIRTs) have recently been regarded as PD pathology modifiers [79]; however, their detailed mechanisms remain elusive.

There are seven types of SIRTs, denoted SIRT1∼7 [86], which are NAD-dependent protein deacetylases and/or ADP-ribosyltransferases that regulate apoptosis, stress resistance, mitochondrial biogenesis, gene expression, and antioxidant defense for maintaining energy metabolism and homeostasis [42]. SIRT1 and SIRT2 are present in the nuclei and cytosol; SIRT3, SIRT4, and SIRT5 are localized in mitochondria; and SIRT6 and SIRT7 are mainly distributed in the nucleus [44,65]. Among them, SIRT1 has been intensively studied for its anti-aging effects [40,73]. Thus, SIRT1 emerges as a therapeutic target for neurodegenerative diseases [26]. Previously, resveratrol (a known natural SIRT1 activator) has been shown to attenuate Parkinsonian mimetics induced by compounds such as 6-hydroxydopamine (6-OHDA), rotenone, and 1-methyl-4-phenyl-1,2,3,6-tetrahydropyridine (MPTP) or its active metabolite 1-methyl-4-phenylpyridinium (MPP^+^) in the mammalian cells [4,45,50], fruit fly [49], and animal [1,34] models of PD. In addition, reduced SIRT1 levels have been found either in the Parkinsonian mimetic-induced PD models [63,69,88] or the frontal cortex of PD patients [76], and several genetic variants in the SIRT1 promoter region have been found in sporadic PD patients [94], further suggesting SIRT1 as a therapeutic target for PD [43].

However, there are still findings that challenge the neuroprotective role of SIRT1. For instance, SIRT1-overexpressed transgenic mice fail to alleviate MPTP-induced loss of nigrostriatal DAergic neurons [37], and SIRT1 deficiency attenuates MPP^+^-induced apoptosis in DAergic cells [66]. Resveratrol's multiple targets and antioxidative nature [8] also question its specificity on SIRT1 activation, although Sinclair's laboratory has shown that SIRT1 activation by resveratrol is mediated through an allosteric mechanism depending on the SIRT1 substrate [31]. Thus, the elucidation of the role of SIRT1 in treating PD is necessary. On the other hand, for the natural and nonspecific SIRT1 activation of resveratrol, synthetic SIRT1 activating compounds (STACs) with greater potency, bioavailability, and solubility have been screened and developed [32]. Among them, SRT1720 has more potency than resveratrol and other derivatives [56]. Originally, it has been synthesized by Sirtris Pharmaceuticals, aiming to treat type 2 diabetes [56] and extend lifespan [57]. Although the direct effect of activating SIRT1 has been questioned previously [62], its biological functions in treating different disease models have been intensively studied. For instance, it has been shown to treat cancer [10], inflammation [33], ischemia [82], hepatitis [96], osteoarthritis [59], and neurodegeneration [6], such as Alzheimer's disease. However, the effect of SRT1720 on PD has rarely been studied.

In addition, although MPTP and 6-OHDA are valuable models for symptomatic therapies for motor assessment in PD, therapeutic approaches tested in these models in many clinical trials have been futile in proving efficacy in identifying disease-modifying therapies [14]. Thus, their suitability as Parkinsonian mimetics should be revisited. Besides, since 5–10% of PD patients are familial [60], other risk factors, such as environment contributing to PD etiology have been intensively studied. Previous literature has suggested a consistent correlation between Parkinson's disease and exposure to pesticides such as paraquat (PQ) and PD [80]. Indeed, PQ resulted in either cell death *in vitro* [19,23] or DAergic neuronal loss and intracellular α-synuclein deposits in the SNpc *in vivo* [53,54], providing an alternative and reasonable Parkinsonian mimicking drug.

Taken together, since our earlier articles have already shown that PQ induces cytotoxicity in human neuroblastoma SH-SY5Y cells [39,90], it was used as a PD model in this study. We also investigated the controversial roles and mechanisms that SIRT1 plays in neurodegenerative diseases and multiple downstream targets, such as the peroxisome proliferator-activated receptor gamma-assisted activating factor-1α (PGC-1α) which regulates mitochondrial function [25], Forkhead-box transcription factor (FOXO) which involves aging [81], and nuclear factor E2-related factor 2 (NRF2) which regulates antioxidative enzyme responses [92], and the roles and mechanisms that SIRT1 play in regulating cytotoxicity [70] and mediating PQ-induced cytotoxicity in human SH-SY5Y cells as a PD model.

## 2 Results

### 2.1 SRT1720-attenuated PQ-induced apoptosis in human SH-SY5Y cells

**Fig. 1:**
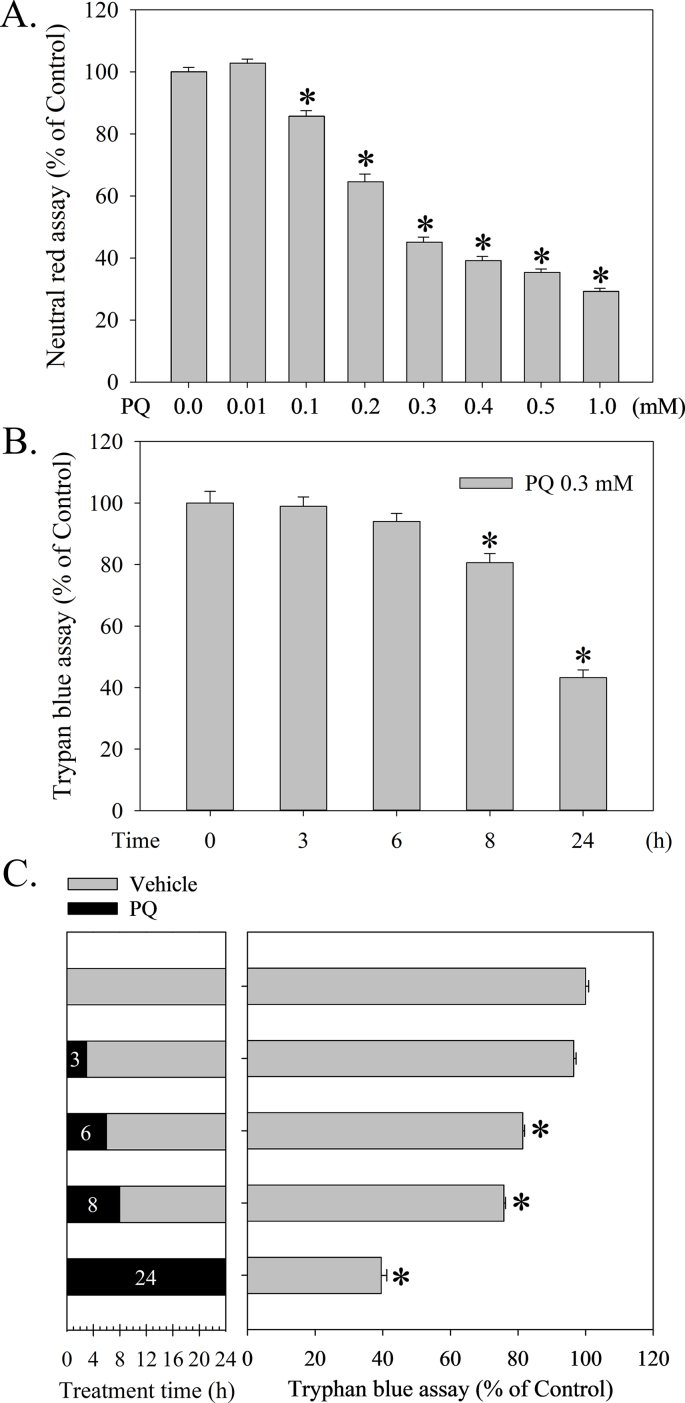
Paraquat (PQ) dose-dependently induced cell death in human neuroblastoma SH-SY5Y cells. (A) SH-SY5Y cells were treated with PQ (0–1 mM) for 24 h, and a neutral red survival assay was then performed to measure viability. (B) Cells treated with 0.3 mM PQ for different intervals (3, 6, 8, and 24 h) were subjected to a trypan blue exclusion assay to measure viability. (C) After PQ treatment for 0, 3, 6, 8, and 24 h, cells were washed once with culturing medium (Vehicle) and then re-loaded with medium and incubated for another 24, 21, 18, 16, and 0 h, respectively. Cells were directly subjected to trypan blue exclusion assay. Viability is represented as a percentage of the results from the neutral red or trypan blue assay compared with controls. Data points represent the mean ± SD of at least three experiments. Differences between groups in raw data were evaluated through one-way ANOVA and considered significant at *p* < 0.05. *p < 0.05, compared to the control group.

**Fig. 2:**
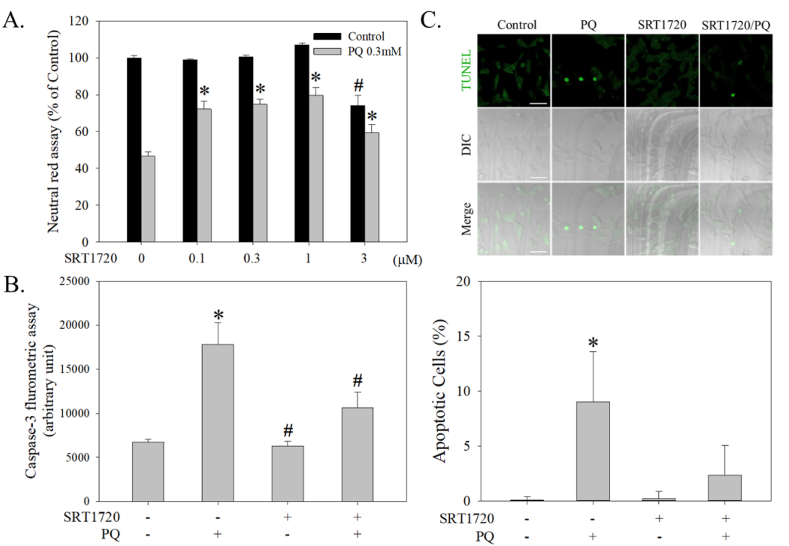
Effects of SRT1720 in attenuating PQ-induced apoptosis in human neuroblastoma SH-SY5Y cells. (A) Cells pretreated with SRT1720 (0–3 μM) for 1 h were treated with or without 0.3 mM PQ for another 24 h. A neutral red survival assay was performed to measure cell viability. Results are a percentage of the neutral red assay results compared to controls. (B) Cells pretreated with 1 μM SRT1720 for 1 h were treated with or without 0.3 mM PQ for another 24 h. Cells were then collected and subjected to caspase 3 activity assay. (C) The TUNEL fluorescence signal was acquired using a confocal microscope, and apoptotic cells were calculated. The bar represents 5 μm. Data points represent the mean ± SD of at least ten frames and 350 cells. Differences among groups were evaluated through one-way ANOVA. *p < 0.05, compared to the control group. #*p* < 0.05, compared to the PQ-treated group. (For interpretation of the references to colour in this figure legend, the reader is referred to the Web version of this article.)

PQ significantly and dose-dependently induced cell death as measured by neutral red survival assay (F_7, 144_ = 400.8, *p* < 0.001) (Fig. 1A). Since the LD50 of PQ is 0.27 mM, we used approximately 0.3 mM for the following experiments. The trypan blue assay showed that PQ treatment began to significantly induce cell death after 8 h (F_4, 12_ = 38.0, *p* < 0.001) (Fig. 1B). However, although PQ treatment for 6 h failed to induce cell death (Fig. 1B), a 6 h PQ treatment followed by a medium replacement experiment for 18 h started inducing cell death significantly (Fig. 1C) (F_4, 13_ = 25.6, *p* < 0.001). In addition, SRT1720 dose-dependently attenuated PQ-induced cell death (Fig. 2A) (F_1, 98_ = 63.7, *p* < 0.001), whereas SRT1720 in a higher dose (≧3 μM) resulted in significant cell death. SRT1720 at 1 μM tended to exert the highest protection against PQ-induced cell death and was therefore adopted in the below-described experiment (Fig. 2A). Furthermore, SRT1720 not only attenuated PQ-induced increased caspase 3 activity (F_3, 23_ = 13.2, *p* < 0.001) (Fig. 2B), but also reduced PQ-induced increased numbers of TUNEL stainings (F_3, 25_ = 25.1, *p* < 0.001) (Fig. 2C).

### 2.2 SRT1720 differentially regulated PQ-mediated expressions of SIRTs

**Fig. 3:**
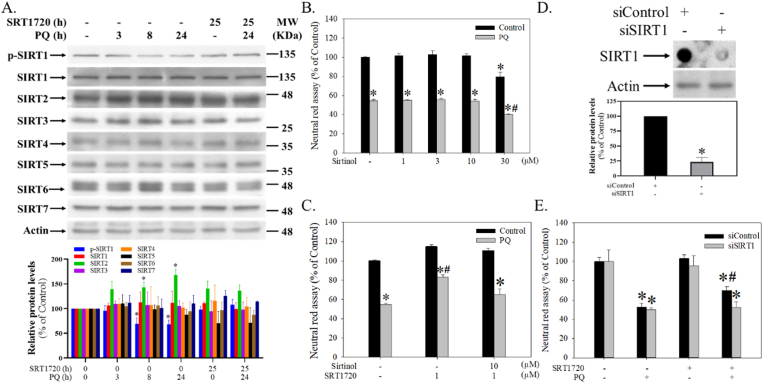
The significance of SIRT1 in SRT1720-mediated protection against PQ-induced cytotoxicity in human neuroblastoma SH-SY5Y cells. (A) Cells pretreated with 1 μM SRT1720 for 1 h were treated with or without 0.3 mM PQ over different intervals. Cells were then harvested and analyzed by Western blotting. The intensities in each group were subdivided by the internal control and normalized to the control group (100%). Data points represent the mean ± SD of three experiments. Differences between groups were evaluated through Kruskal-Wallis analysis of variance on ranks with the Student-Newman-Keuls test and considered significant at *p* < 0.05. *p < 0.05, compared to the control group. (B) After pretreatment with different dosages of sirtinol for 1 h, cells were treated with or without 0.3 mM PQ for another 24 h and then subjected to a neutral red survival assay. (C) After pre-inhibition of sirtinol for 30 min, cells were treated with SRT1720 for 1 h and then followed by 0.3 mM PQ treatment for another 24 h. Cells were subjected to a neutral red survival assay. (D) After *SIRT1* silence, cells growing in 6-well plates were subjected to dot blot and Western blot analyses. The relative intensities of SIRT1 were subdivided by the internal control and normalized to the siControl group (100%). Data points represent the mean ± SD of at least three experiments. Differences between groups were evaluated through t-tests and considered significant at *p* < 0.05. (E) After *SIRT1* silencing, cells growing in 96-well plates were subjected to a survival assay. Cell viability represents a percentage of neutral red assay results compared to controls. Data points represent the mean ± SD of at least three experiments. Differences between groups in raw data were evaluated through two-way ANOVA with the Student-Newman-Keuls tests and considered significant at *p* < 0.05. *p < 0.05, compared to the control group. #*p* < 0.05, compared to the PQ-treated group. (For interpretation of the references to colour in this figure legend, the reader is referred to the Web version of this article.)

The expressions of SIRTs in SH-SY5Y cells were differentially regulated after PQ and SRT1720 treatments (Fig. 3A). Among them, the levels of SIRT1 (H = 5.1, df = 5, *p* = 0.4), SIRT3 (H = 0.8, df = 5, *p* = 2.1), SIRT4 (H = 6.7, df = 5, *p* = 0.2), SIRT5 (H = 7.0, df = 5, *p* = 0.2), SIRT6 (H = 5.4, df = 5, *p* = 0.4), and SIRT7 (H = 8.8, df = 5, *p* = 0.1) were not significantly altered by PQ with or without SRT1720 pretreatment (Fig. 3A). However, SIRT2 was significantly elevated by PQ (H = 13.4, df = 5, *p* < 0.05) and partially attenuated by SRT1720 pretreatment (Fig. 3A). Although PQ did not result in an altered expression of SIRT1, it significantly suppressed the phosphorylation of SIRT1 (*p*-SIRT1) (H = 12.8, df = 5, *p* < 0.05), which was then be reversed by SRT1720 pretreatment (Fig. 3A).

### 2.3 Blockade of SIRT1 attenuated the protection of SRT1720 against PQ-induced cell death in human SH-SY5Y cells

Sirtinol (a known SIRT1 inhibitor) ranging from 1–10 μM exerted no significant effects on cells treated with or without PQ (Fig. 3B) (F_1, 20_ = 274.9, *p* < 0.001). However, sirtinol at 30 μM induced significant cell death and worsened the toxicity induced by PQ (Fig. 3B). In addition, 10 μM sirtinol significantly attenuated SRT1720 protection against PQ-induced cell death (Fig. 3C) (F_1, 12_ = 68.9, *p* < 0.001). Further, *SIRT1* silencing not only suppressed *SIRT1* expression (t = 17.8, df = 4, *p* < 0.0001) (Fig. 3D, upper and lower panels), but also attenuated protection by SRT1720 (Fig. 3E) (F_1, 16_ = 125.5, *p* < 0.001).

### 2.4 SRT1720 attenuated PQ-induced ROS formation and mitochondrial dysfunction in human SH-SY5Y cells

**Fig. 4:**
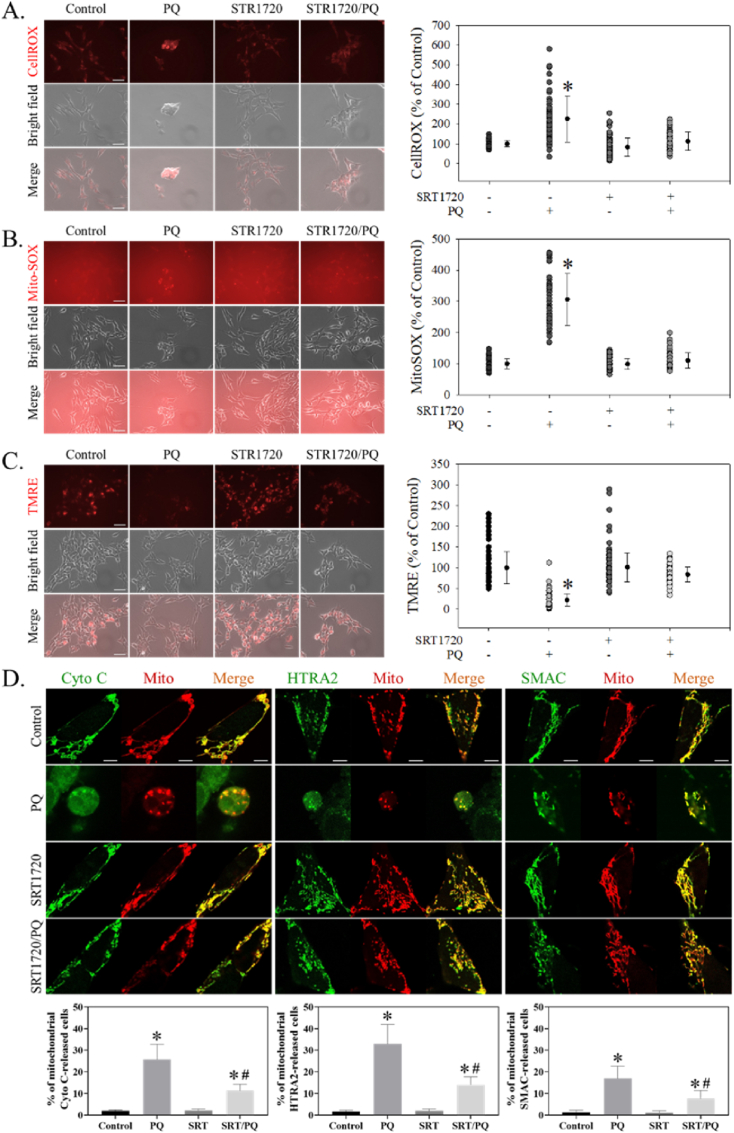
SRT1720 attenuated PQ-induced ROS formation and mitochondrial dysfunction in human neuroblastoma SH-SY5Y cells. After pretreatment with 1 μM SRT1720 for 1 h, cells were treated with or without 0.3 mM PQ for another 24 h. Cells were then stained with (A) CellROX Orange, (B) MitoSOX, and (C) TMRE and subjected to image acquisition. Cell fluorescence intensities were quantitated by ImageJ and represented as a percentage of the results compared to controls. The bar represents 5 μm. Data points represent the mean ± SD of at least 100 cells. Differences among groups were evaluated through one-way ANOVA on ranks. *p < 0.05, compared to the control group. (D) After co-transfection with plasmids (pDsRed2-Mito/pGFP-Cytochrome (Cyto) C, pDsRed2-Mito/pGFP-HtrA2, or pDsRed2-Mito/pSmac-GFP) for 24 h, cells were pretreated with 1 μM SRT1720 (SRT) for 1 h and then received 0.3 mM PQ treatments for another 24 h. Cells were fixed and subjected to confocal microscopy analysis. The bar represents 5 μm. In each treatment, at least 15 transfected cells in every 3 slides were randomly acquired, and the percentages of cells with released mitochondrial proapoptotic factors were counted and calculated. Differences among groups were evaluated through a Kruskal-Wallis analysis of variance on ranks. *p < 0.05 compared to the PQ-treated group. (For interpretation of the references to colour in this figure legend, the reader is referred to the Web version of this article.)

SRT1720 significantly attenuated PQ-induced increased the fluorescence intensities of CellROX Orange (H = 148.7, df = 3, *p* < 0.001) (Fig. 4A) and MitoSOX (H = 215.2, df = 3, *p* < 0.001) (Fig. 4B). SRT1720 also significantly reversed the PQ-induced decreased fluorescence intensity of TMRE (H = 319.6, df = 3, *p* < 0.001) (Fig. 4C). Further, SRT1720 attenuated PQ-induced mitochondrial releases of apoptosis-inducing factors (Fig. 4D) such as cytochrome C (H = 13.0, df = 3, *p* < 0.01), HTRA2 (H = 12.8, df = 3, *p* < 0.01), and SMAC (H = 12.9, df = 3, *p* < 0.01).

### 2.5 SRT1720 attenuated PQ-induced down-regulation of PGC-1α level in human SH-SY5Y cells

**Fig. 5:**
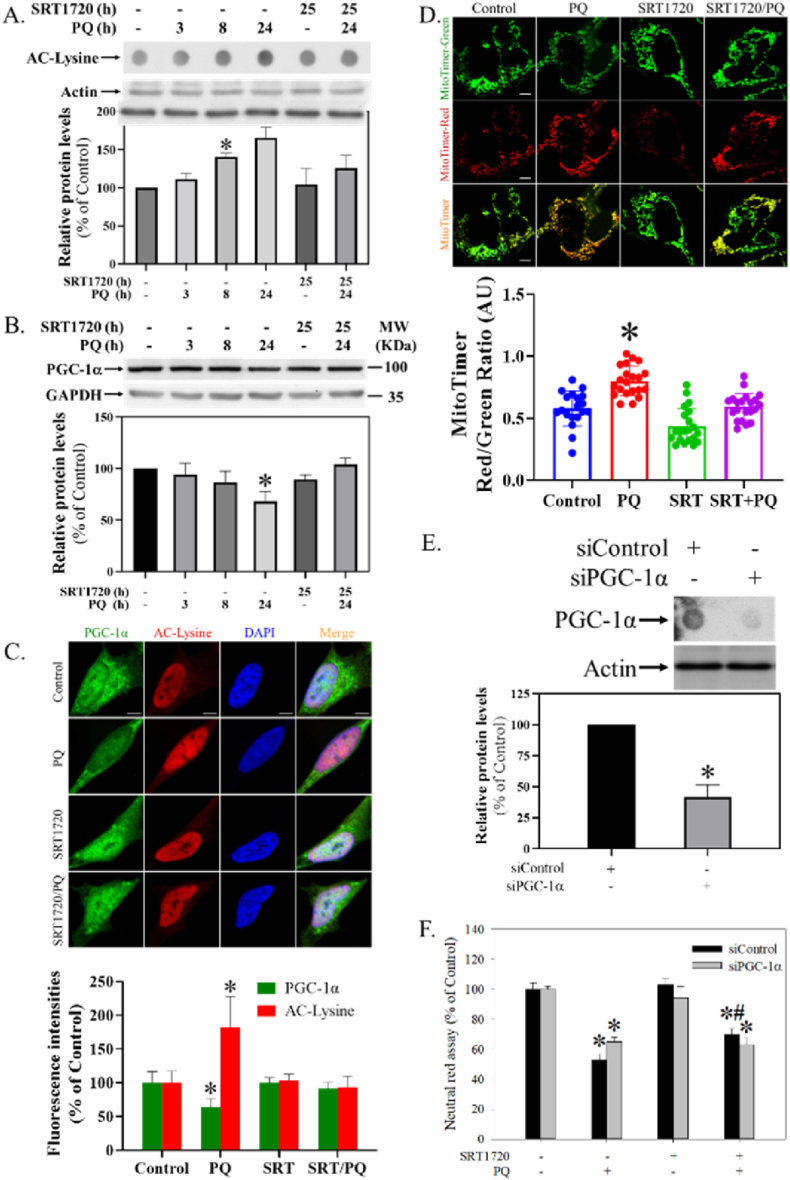
Effect of SRT1720 in modulating PQ-induced alterations of protein acetylation and PGC-1α function. Cells pretreated with 1 μM SRT1720 (SRT) for 1 h were treated with or without 0.3 mM PQ over different intervals. Cells were harvested and analyzed by (A) dot blot assay, (B) Western blotting, and (C) ICC. The intensities in each group were divided by the internal control and normalized to the control group (100%). Data points represent the mean ± SD of three experiments. Differences between groups were evaluated through Kruskal-Wallis analysis of variance on ranks with the Student-Newman-Keuls test and considered significant at *p* < 0.05. *p < 0.05, compared to the control group. (D) After pMitoTimer transfection for 24 h, cells were treated with 1 μM SRT and 0.3 mM PQ as described above. The ratio of red and green fluorescence intensities of mitochondria was quantitated by ImageJ. Differences between groups were evaluated through one-way ANOVA with the Student-Newman-Keuls test and considered significant at *p* < 0.05. *p < 0.05 compared to the control group. The bar represents 5 μm. (E) Cells with *PGC-1α* silencing were subjected to dot blot and Western blot analyses. The intensities of PGC-1α were divided by the internal control and normalized to the siControl group (100%). Data points represent the mean ± SD of three experiments. Differences between groups were evaluated through t-tests and considered significant at *p* < 0.05. (F) After *PGC-1α* silencing, cells growing in 96-well plates were subjected to a survival assay. Cell viability was represented as a percentage of the neutral red assay results compared with controls. Data points represent the mean ± SD of at least three experiments. Differences between groups in raw data were evaluated through two-way ANOVA with the Student-Newman-Keuls test and considered significant at *p* < 0.05. *p < 0.05, compared to the control group. #*p* < 0.05, compared to the PQ-treated group. (For interpretation of the references to colour in this figure legend, the reader is referred to the Web version of this article.)

In the dot blot assay, SRT1720 significantly and time-dependently attenuated PQ-induced increased protein acetylations (Fig. 5A) (H = 14.1, df = 5, *p* < 0.05). In the Western blot analysis, SRT1720 significantly reversed the PQ-induced down-regulation of PGC-1α levels (Fig. 5B) (H = 12.8, df = 5, *p* < 0.05). In the immunocytochemistry assay, SRT1720 attenuated the PQ-induced decreased PGC-1α expression and increased nuclear acetylation (Fig. 5C) (H = 21.9, df = 3, *p <* 0.001). In the MitoTimer assay, SRT1720 attenuated the PQ-induced increase in the ratio of red/green fluorescence intensities (F_3, 78_ = 28.7, p < 0.001) (Fig. 5D). Further, *PGC-1α* silencing not only suppressed *SIRT1* expression (t = 10.1, df = 4, *p* < 0.001) (Fig. 5E), but also attenuated the protection of SRT1720 (Fig. 5F) (F_1, 16_ = 92.6, *p* < 0.001).

### 2.6 SRT1720 regulated PQ-mediated alterations of KEAP1/NRF2 levels in human SH-SY5Y cells

**Fig. 6:**
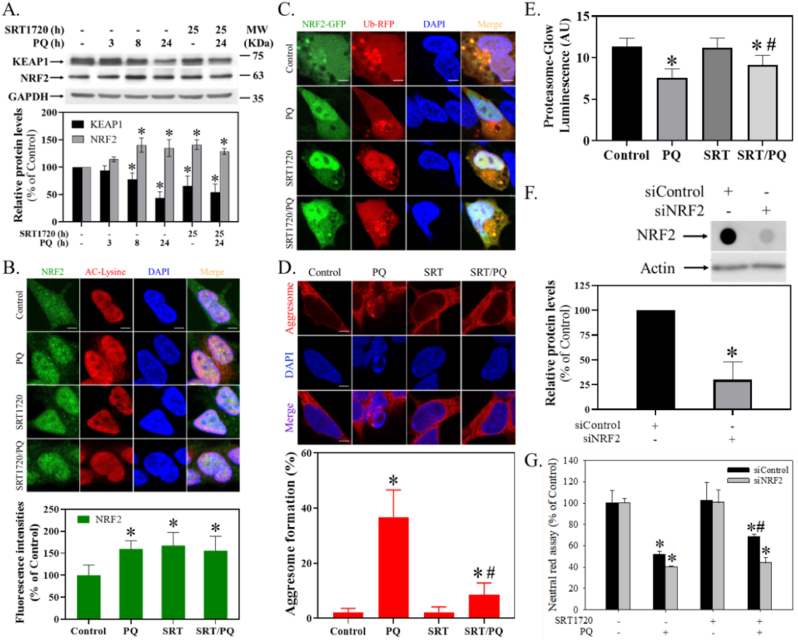
Effect of SRT1720 on the PQ-mediated KEAP1/NRF2 pathway. Cells pretreated with 1 μM SRT1720 (SRT) for 1 h were treated with or without 0.3 mM PQ. Cells were subjected to (A) Western blotting and (B) ICC. The intensities were divided by the internal control and normalized to the control group (100%). Data points represented the mean ± SD of three experiments. Cells transfecting with pNRF2-GFP and pUb-RFP (6:4) were pretreated with 1 μM SRT and 0.3 mM PQ for 24 h, and a confocal microscope acquired the images (C). Furthermore, after 1 μM SRT and 0.3 mM PQ treatment as described above, cells were subjected to (D) aggresome and (E) proteasome activity assay. The bar represents 5 μm in all imaging. The percentage of red punctated aggresome of at least 30 cells in every 5 fields was counted. The luminescence intensities in the arbitrary unit (AU) were calculated. Data points represent the mean ± SD of three experiments. Differences among groups were evaluated through one-way ANOVA with the Student-Newman-Keuls test and considered significant at *p* < 0.05. *p < 0.05, compared to the control group. #*p* < 0.05, compared to the PQ-treated group. Alternatively, after *NRF2* silencing, cells were subjected to (F) dot blot, Western blot analysis, and (G) survival assay. The relative intensities of NRF2 were divided by the internal control and normalized to the siControl group (100%). Data points represent the mean ± SD of three experiments. Differences between groups were evaluated through t-testing and considered significant at *p* < 0.05. Cell viability was represented as a percentage of the neutral red assay results compared to controls. Differences between groups of raw data were evaluated through two-way ANOVA with the Student-Newman-Keuls test and considered significant at *p* < 0.05. *p < 0.05, compared to the control group. #*p* < 0.05, compared to the PQ-treated group. (For interpretation of the references to colour in this figure legend, the reader is referred to the Web version of this article.)

PQ and SRT1720 both significantly reduced the level of KEAP1 (H = 14.3, df = 5, *p* < 0.05) (Fig. 6A) and increased the level of NRF2 (Fig. 6A) (H = 13.6, df = 5, p < 0.05). In the immunocytochemistry assay, PQ and SRT1720 elevated the fluorescence intensity (H = 20.1, df = 3, p < 0.001) and nucleus translocation of NRF2 (Fig. 6B). In the transient transfection assay, overexpressed NRF2-GFP (green fluorescence protein) tended to dissociate from the overexpressed Ub-RFP (red fluorescence protein), and translocated to the nucleus after PQ and SRT1720 treatment (Fig. 6C). Alternatively, PQ significantly induced the punctated form of aggresomes, which SRT1720 attenuated (Fig. 6D) (H = 15.5, df = 3, p < 0.01). Further, SRT1720 also significantly reversed PQ-induced decreased proteasomal activity (Fig. 6E) (F_3, 8_ = 8.1, *p* < 0.01). Further, *NRF2* silencing not only suppressed *NRF2* expression (t = 6.7, df = 4, *p* < 0.01) (Fig. 6F), but also attenuated the protection of SRT1720 from PQ-induced cytotoxicity (F_1, 16_ = 7.0, *p* < 0.05) (Fig. 6G).

### 2.7 SRT1720 reversed PQ-mediated decreased activities of antioxidant enzymes in human SH-SY5Y cells

**Fig. 7:**
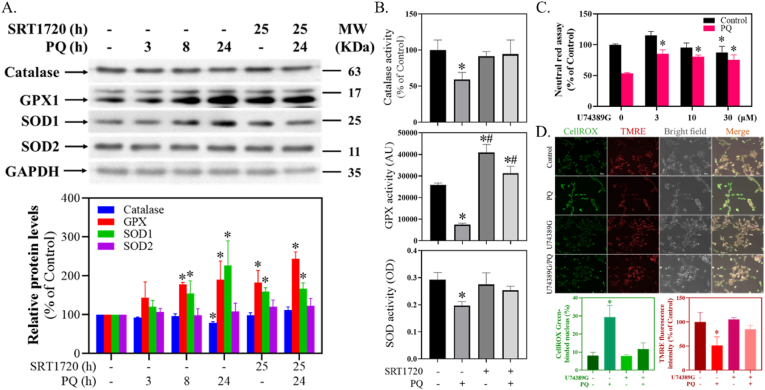
Effect of SRT1720 on PQ-mediated antioxidative enzymes and U74389G on PQ-induced cytotoxicity. (A) Cells pretreated with 1 μM SRT1720 for 1 h were treated with or without 0.3 mM PQ and subjected to Western blotting. The intensities in each group were divided by the internal control and normalized to the control group. Data points represent the mean ± SD of three experiments. Differences between groups were evaluated through one-way ANOVA with the Student-Newman-Keuls test and considered significant at *p* < 0.05. *p < 0.05, compared to the control group. (B) As described above, cells treated with SRT1720 and PQ were harvested for enzyme activity assays. Data points represent the mean ± SD of three experiments. Differences between groups were evaluated through one-way analysis of variance with the Student-Newman-Keuls test and considered significant at *p* < 0.05. *p < 0.05, compared to the control group. (C) After 1 h U74389G pretreatment, cells were treated with 0.3 mM PQ for 24 h and subjected to a neutral red survival assay. Cell viability was represented as a percentage of the neutral red assay results compared to controls. Data points represent the mean ± SD of three experiments. Differences between groups in raw data were evaluated through two-way ANOVA with the Student-Newman-Keuls test and considered significant at *p* < 0.05. *p < 0.05, compared to the control group. (D) After 3 μM U74389G pretreatment for 1 h and 0.3 mM PQ treatment for 24 h, cell images were randomly acquired. The bar represents 50 μm. The percentages of the CellROX Green Reagent-bonded nucleus and the TMRE fluorescence intensity were quantitated and normalized with respect to the control group. Data points represent the mean ± SD of three experiments. Differences between groups were evaluated through one-way ANOVA with the Student-Newman-Keuls test and considered significant at *p* < 0.05. *p < 0.05, compared to the control group. (For interpretation of the references to colour in this figure legend, the reader is referred to the Web version of this article.)

SRT1720 significantly attenuated PQ-induced decreased levels of catalase (H = 13.4, df = 5, *p* < 0.05) and increased levels of GPX (H = 12.2, df = 5, *p* < 0.05) and SOD1 (H = 13.3, df = 5, *p* < 0.05) (Fig. 7A). However, SRT1720 and PQ did not affect the level of SOD2 (H = 6.8, df = 5, *p* = 0.23) (Fig. 7A). SRT1720 further significantly reversed the PQ-induced decreased activity of catalase (F_3,8_ = 5.6, *p* < 0.05), GPX (F_3,8_ = 93.5, *p* < 0.05) and SOD (F_3,8_ = 10.7, *p* < 0.05) (Fig. 7B).

### 2.8 U74389G attenuated PQ-induced cytotoxicity in human SH-SY5Y cells

U74389G significantly attenuated PQ-induced cell death (F_7, 15_ = 32.4, *p* < 0.001) (Fig. 7C). U74389G also significantly attenuated PQ-induced increased nucleus-aggregated CellROX Green (H = 9.4, df = 3, *p* < 0.05) (Fig. 7D, upper and lower panels) and decreased TMRE fluorescence intensities (H = 9.8, df = 3, *p* < 0.05) (Fig. 7D, upper and lower panels).

### 2.9 SRT1720 attenuated PQ-mediated decreased TH staining and behavior performance in mice

SRT1720 significantly attenuated PQ-induced decreased stainings of TH (H = 10.1, df = 3, *p* < 0.05) and PGC-1α (H = 10.1, df = 3, *p* < 0.05) in midbrain SNpc DAergic neurons. SRT1720 pretreatment significantly reversed PQ-induced increased number of slips in the balance beam test (F_3,8_ = 48.7, *p* < 0.0001).

## 3 Discussion

Our prior work has mainly focused on the role of mitochondria in mediating PQ-induced cytotoxicity in rat pheochromocytoma PC12 cells [29,30] and human neuroblastoma SH-SY5Y cells [39]. This study examined the mechanism and therapeutic potential of SRT1720 acting through SIRT1 to mediate PQ-induced cytotoxicity. Since our pilot study has already profiled the expression of FOXO1 and found no difference between SRT1720-and/or PQ-treated groups (Supplementary Fig. 1A), and a FOXO relocator (LOM612) failed to prevent PQ-induced cell death (Supplementary Fig. 1B), the functional role of FOXO1 will no longer be studied by us.

### 3.1 SRT1720 and PQ-induced apoptosis in human SH-SY5Y cells

We reproduced PD-induced cytotoxicity in human neuroblastoma SH-SY5Y cells as an *in vitro* model of PD (Fig. 1A). Further, we have determined that PQ treatment for 8 h but not 6 h started to induce significant cell death (Fig. 1B). However, although PQ treatment for 6 h did not induce significant cell death, this period tended to be a threshold time point having no reversal (Fig. 1C). We also observed that PQ induced apoptotic cell death by showing increased caspase 3 activity and TUNEL staining (Fig. 2B and C). Based on this *in vitro* PD model, SRT1720 has, for the first time, been found to exert protection against PQ-induced apoptotic death in human neuroblastoma SH-SY5Y cells (Fig. 2A, B, and 2C), consistent with the recent finding that resveratrol protects against PQ-induced cytotoxicity in PC12 cells [95].

### 3.2 SRT1720 and PQ-mediated expressions of SIRTs

We next examined the effects of SRT1720 and PQ on SIRTs. While 0.3 mM PQ did not alter the expressions of SIRT1, SIRT3, SIRT4, SIRT5, SIRT6, and SIRT7, we were surprised to find that the level of SIRT2 increased and the phosphorylation of SIRT1 decreased (Fig. 3A), which could be attenuated by SRT1720. Because SIRT2 has been shown to increase α-synuclein aggregation, exacerbate oxidative stress damage, decrease microtubule stability, and aggravate neuroinflammation in different PD models [48], the SRT1720-mediated suppression of PQ-induced SIRT2 expression could be a protective mechanism. However, another study shows that SIRT2 enhances MPTP-induced nigrostriatal damage [46], implying an obscure role of SIRT2 in PD pathogenesis. We thus validated the significance of SIRT2 and found that a known SIRT2 inhibitor (AGK2) failed to prevent PQ-induced cytotoxicity (Supplementary Fig. 2), indicating a dispensable role of SIRT2 in this system. Accordingly, since PQ tended to suppress the activity of SIRT1, which was reversed by SRT1720 (Fig. 3A), the significance of SIRT1 was examined. The pharmacological inhibition of SIRT1 by sirtinol (Fig. 3C) and genetic silencing of *SIRT1* (Fig. 3E) both attenuated the protection of SRT1720, indicating that SRT1720 might mediate through SIRT1 to prevent PQ-induced toxicity and be consistent with a previous study that also shows SIRT1 prevents PQ-induced injury in mouse type II alveolar epithelial cells [16].

### 3.3 SRT1720 and PQ-induced cytotoxicity

Alternatively, since the redox-cycling and enzymatic reactions of pro-oxidant PQ [18] resulting in oxidative stress and mitochondrial dysfunction have been regarded as the main cytotoxic mechanisms [3,58], we examined and confirmed that SRT1720 attenuates PQ-induced oxidative stress (Fig. 4A) and mitochondrial dysfunction, including increased mitochondrial superoxide formation (Fig. 4B) and decreased membrane potential (Fig. 4C). Since PQ-induced mitochondrial dysfunction renders increased mitochondrial membrane permeability and subsequent proapoptotic factor release [39], we confirmed that PQ does induce the release of pro-apoptosis-inducing factors (including cytochrome C, HTRA2, and SMAC) (Fig. 4D), consistent with our [39] and others' previous findings [23]. Thus, SRT1720 preventing the release of these factors may strengthen its protection against PQ-induced mitochondrial dysfunction. These effects coincide with the functions of SIRT1 in regulating oxidative stress and mitochondrial dysfunction in PD models [43,76], further supporting that SRT1720 mediates through SIRT1 to prevent PQ-induced toxicity.

### 3.4 SRT1720, PGC-1α, and PQ-induced cytotoxicity

Since PQ tended to reduce SIRT1 activity as demonstrated by decreased phosphorylation (Fig. 3A), we examined whether PQ would result in increased acetylation. As expected, acetylations were elevated during PQ intoxication (Fig. 3A), consistent with previous studies that show PQ promotes histone acetylation in DAergic cells [61] and imbalanced acetylation contributes to PD pathogenesis [67]. Again, whether PQ-induced impaired deacetylation plays a role in regulating cell death still needs further investigation. The protection of SRT1720 in reducing PQ-induced elevated acetylations (Fig. 3A) may further confirm that SRT1720 promotes SIRT1 activity.

SIRT1 prevents oxidative stress and mitochondrial dysfunction in many neuronal disease models [43], such as autistic spectrum disorder [5], intracerebral hemorrhage [97], ischemia/reperfusion [41], status epilepticus [87], and PD [13], through the PGC-1α pathway. Therefore, we examined and found that PQ resulted in decreased PGC-1α expression (Fig. 5B and C), which is partly compatible with a previous study [13]. PGC-1α is a well-known transcriptional coactivator and master regulator of mitochondrial biogenesis [20], and we did determine that SRT1720 increased PGC-1α promoter activity (Supplementary Fig. 3). Subsequently, we applied a novel plasmid (pMitoTimer) for real-time monitoring of mitochondrial biogenesis [38] and found that PQ did impair mitochondrial biogenesis (Fig. 5D), possibly owing to PQ-induced decreased PGC-1α level (Fig. 5B). Thus, SRT1720 mediation through PGC-1α-dependent mitochondrial biogenesis could be a protective mechanism in preventing PQ cytotoxicity, which is partly consistent with a previous study [21]. Further, to test the significance of PGC-1α, it was silenced (Fig. 5E) and the protection of SRT1720 was subsequently attenuated (Fig. 5F), supporting SRT1720 having a key role in the PGC-1α pathway for attenuating PQ-induced cytotoxicity.

### 3.5 SRT1720, NRF2, and PQ-induced cytotoxicity

NRF2 as a pleiotropic transcription factor has been recognized as a master regulator of antioxidant cellular response and is also involved in regulating numerous biological functions that include survival, autophagy, proteostasis, metabolism, and differentiation [28]. Since we previously found that the SIRT1-mediated NRF2 pathway [16] and *NRF2* overexpression [89] can prevent PQ-induced cell injury, we next examined the involvement of NRF2 during PQ intoxication. Normally, NRF2 is bound majorly to KEAP1, which promotes NRF2 ubiquitination and subsequently the proteasomal degradation of NRF2 [93]. However, during cell stressing, such as exposure to electrophile toxicants, chemopreventive molecules, or oxidative stress, the cysteine or phosphorylation modification of KEAP1 dissociates it from NRF2, leading to NRF2 nucleus translocation. Thus, in addition to the possibility of KEAP1 modification by PQ-induced-oxidative stressing (Fig. 4), we also observed that PQ and SRT1720 decreased KEAP1 levels (Fig. 6A). Since KEAP1 degradation has been found during autophagy in the maintenance of redox homeostasis [77], and PQ [22,39] (Supplementary Figs. 4A and 4B) and SRT1720 [51] have both also been shown to induce autophagy, PQ- or SRT1720-induced autophagy may contribute to KEAP1 degradation that subsequently results in NRF2 elevation (Fig. 6A), dissociation (Fig. 6B), and nucleus translocation (Fig. 6B and C).

To further validate the above mechanisms, we directly co-transfected pNRF2-GFP and pUb-RFP to demonstrate decreased NRF2 ubiquitination (Fig. 6C). Further, since the ubiquitin-proteasome system (UPS) and autophagy are major systems for protein degradation, we detected the increase of aggresomes (Fig. 6D) and decrease in proteasomal activity (Fig. 6E) during PQ intoxication, indicating PQ-induced proteasomal dysfunction consistent with a previous study [91]. These results may well explain that PQ-induced NRF2 elevation and nucleus translocation may be due to increased KEAP1 degradation and decreased ubiquitination and proteasomal activity. However, although we have shown that KEAP1 degradation could be the mechanism that PQ and SRT1720 have in elevating NRF2, another p62-dependent noncanonical pathway [35] in regulating KEAP1 activity might also exist in this system. Accordingly, when phosphorylated, p62 competitively binds to KEAP1 to activate NRF2. Indeed, we have shown for the first time that it was PQ but not SRT1720 that elevated the protein level and phosphorylation of p62 (Supplementary Fig. 5). Thus, p62 may be an alternative pathway for PQ to activate NRF2. However, further study is required to answer this question. Lastly, *NRF2* was silenced to identify the significance of NRF2 during PQ and SRT1720 treatment (Fig. 6E), resulting in only the protective effect of SRT1720 being attenuated (Fig. 6F), thus supporting NRF2 being an important signaling event in protecting SRT1720.

### 3.6 SRT1720, PQ, and antioxidative enzymes

NRF2 is regarded as a master mediator for cellular redox homeostasis [47]. When translocating to the nucleus and combining with a small musculoaponeurotic fibrosarcoma (sMaf), it binds to the promoter regions of antioxidant response elements (AREs) to stimulate the expressions of antioxidants and detoxification enzymes [36]. These enzymes include heme oxygenase (HO), catalase, GPX, and SOD [83]. We thus examined the expressions of these enzymes during PQ and SRT1720 treatments. Indeed, PQ and SRT1720 significantly elevated the levels of GPX1 and SOD1 (Fig. 7A). However, PQ decreased the catalase level, which was reversed by SRT1720 (Fig. 7A). Since PGC-1α also regulates the expressions of antioxidative enzymes [61,72], it is possible that SRT1720-mediated PGC-1α expression may counteract such effects. This result also revealed a close interaction between NRF2 and PGC-1α signaling pathways [15,24]. Further, other related enzymes, including HO1, NADPH quinone dehydrogenase 1 (NQO1), thioredoxin (TXN), thioredoxin reductase (TXNRD), and peroxiredoxin (PRDX), were also found to be elevated during PQ intoxication (Supplementary Figs. 6 and 7). These findings might be explained by the NRF2 nucleus translocation, which is compatible with other research [17,75,78].

However, it is perplexing why PQ and SRT1720 both elevated NFR2 and some antioxidative enzyme levels, but only PQ was toxic. Thus, the activities of several typical enzymes were analyzed, revealing that catalase, GPX, and SOD were all decreased by PQ treatment (Fig. 7B), consistent with a previous article [27]. As expected, SRT1720 counteracted these events (Fig. 7B). Currently, although the mechanisms of these decreased enzymatic activities were unclear, we have further observed that PQ increased not only the original but also the oxidized forms of PRDX1/2 levels (Supplementary Fig. 7), suggesting the oxidized inactivation of these enzymes. Such compensatory effects that turn out to be futile could also be found in our previous articles [11,12]. However, further investigations are required to answer this question.

### 3.7 U74389G, PQ, and oxidative stress

A known antioxidant, U74389G (Lazaroid), was applied to estimate the significance of PQ-induced oxidative stress. U74389G exerted protection against PQ-induced cell death (Fig. 7C). This result was similar to research that used SOD/catalase mimetics as the neuroprotective agent for preventing PQ-mediated DAergic neuron death in the SNpc [67]. U74389G also prevented PQ-induced impaired mitochondrial membrane potential and oxidative stress (Fig. 7D), indicating that coping with PQ-induced oxidative stress might be important for cell survival. Alternatively, consistent with our *in vitro* findings (Fig. 6B and C), nucleus stainings of NRF2 of postmortem PD brains have also been found in the nigral DAergic neuron [68]. Since our *in vitro* PD model and other PD patients' brains have found NRF2 nucleus translocation, the NRF2-derived antioxidative enzymes with low activities may fail to cope with free radicals resulting in oxidative stress formation and subsequent cell death. Therefore, the NRF2 pathway has been regarded as a therapeutic target in treating PD [9,84].

### 3.8 SRT1720, PQ, and SNpc TH

**Fig. 8:**
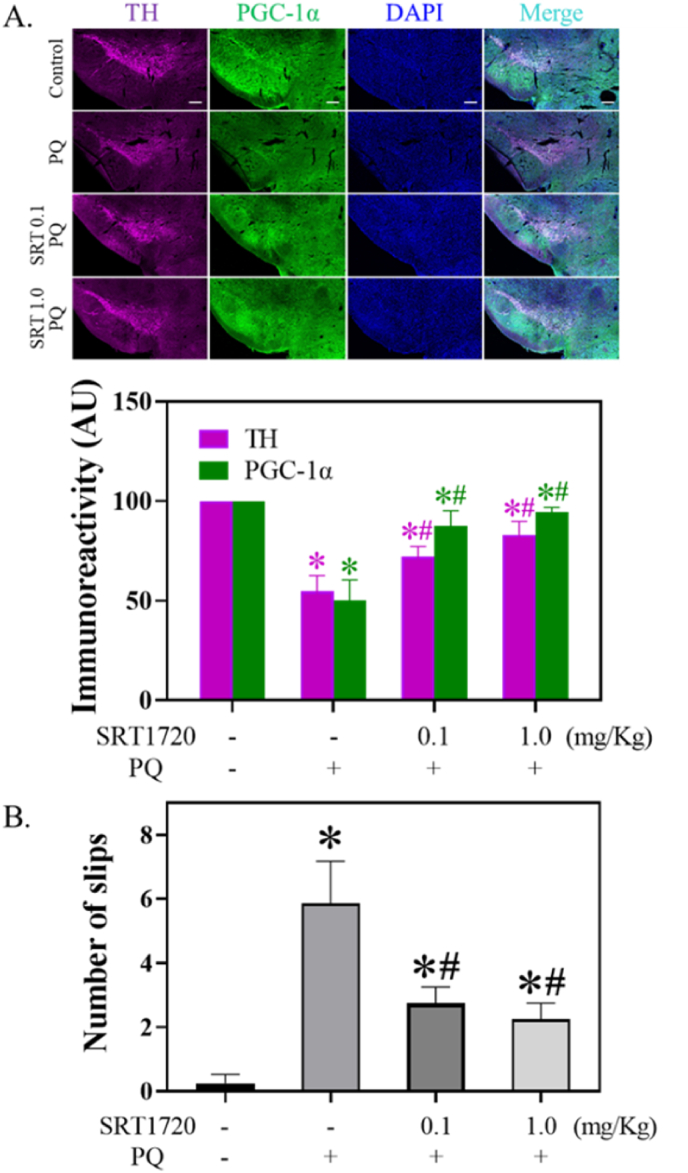
Effect of SRT1720 on PQ-mediated SNpc TH and PGC-1α immunoreactivities and behavioral performance. (A) After SRT1720 and/or PQ treatments, mice were sacrificed through anesthetization, perfusion, fixation, and decapitation. Brain sections were subjected to IHC. The bar represents 200 μm. The immunoreactivities of TH and PGC-1α in SNpc were quantitated by ImageJ. Data represent the means ± S.D. from at least three mice in each group. Differences between groups were evaluated through Kruskal-Wallis analysis of variance on ranks with the Student-Newman-Keuls test and considered significant at *p* < 0.05. *p < 0.05, compared to the control group. #*p* < 0.05, compared to the PQ-treated group. (B) Before sacrificing, a balance beam test was applied to the mice to test their behavioral performance. The number of slips was counted. Data points represent the mean ± SD of at least three experiments. Differences between groups of raw data were evaluated through one-way ANOVA with the Student-Newman-Keuls test and considered significant at *p* < 0.05. *p < 0.05, compared to the control group. #*p* < 0.05, compared to the PQ-treated group.

To validate the protection of SRT1720 against PQ-induced cytotoxicity within *in vitro* to *in vivo* models, SRT1720 was applied to the mouse model of PD by treating with PQ. As expected, SRT1720 reversed the PQ-induced decreased TH and PGC-1α of DA neurons in the midbrain SNpc (Fig. 8A), consistent with our *in vitro* data (Fig. 5B and C). We also observed that SRT1720 attenuates PQ-induced decreased behavioral performance (Fig. 8B), confirming protection by SRT1720 in PQ-treated mice. Thus, it is possible that SRT1720 may mediate through PGC-1α to prevent PQ-induced decreased SNpc TH levels and motor function. However, further work is required to answer this question.

Taken together, we have shown for the first time that SRT1720, as a SIRT1 activator, can prevent PQ-induced cytotoxicity in human neuroblastoma SH-SY5Y cells. The protective mechanisms *in vitr*o involve the regulation of PGC-1α, which invigorates mitochondrial function, and NRF2, which elevates antioxidative enzyme activities, to cope with PQ-induced cellular dysfunction (such as oxidative stress) and subsequently prevent cell death. SRT1720 further preserved midbrain SNpc DAergic TH and PGC-1α levels and motor function in our i*n vivo* PD model, suggesting SRT1720-or SIRT1-targeting drugs as novel therapeutics for treating PD [43].

## 4 Methods

### 4.1 Reagents and kits

Reagents were purchased from Sigma (St. Louis, MO, USA) except otherwise specified. Goat serum (GS) was purchased from Jackson ImmunoResearch Laboratories (West Grove, PA, USA). Dulbecco's modified Eagle's medium (DMEM) and fetal bovine serum (FBS) were purchased from HyClone (Logan, UT, USA). Lipofectamine 2000, fluorescent dyes (such as tetramethylrhodamine ethyl ester (TMRE), Mito-SOX™, and CellROX™ Green and Orange Reagents), and second antibodies were purchased from Thermo Fisher Scientific Inc. (Waltham, MA, USA). SRT1720 (A10862) and sirtinol (A12226) were purchased from Adooq BioScience (Irvine, CA, USA). Anti-phosphorylated (p)-SIRT1(2314), -SIRT1 (9475), -SIRTT2 (12650), -SIRTT3 (5490), -SIRTT5 (8782), -SIRTT6 (12486), and -SIRTT7 (5360) antibodies were purchased from Cell Signaling (Beverly, MA, USA). Anti-SIRT4 (BS7357R) antibody was purchased from Bioss Antibodies (Woburn, MA, USA). Anti-Acetyl Lysine (ab80178), -glutathione peroxidase (GPX; ab108429), -NRF2 (ab31163), -superoxide dismutase 1 (SOD1; ab13498), -superoxide dismutase 2 (SOD2; ab13533), and -tyrosine hydroxylase (TH; ab76442) antibodies were purchased from Abcam (Cambridge, UK). Anti-PGC-1α (NBP1-04676) antibody was purchased from Novus Biologicals (Centennial, CO, USA). Anti-GAPDH (60004-1-Ig) and -KEAP1 (10503-2-AP) antibodies were purchased from Proteintech (Rosemont, IL, USA). All plasmids except pDsRed-Mito (632421; expressing as a red fluorescent mitochondrial marker, purchased from Clontech, Mountain View, CA, USA) were purchased from Addgene (Cambridge, MA, USA). siGENOME SMART pool siRNAs and DharmaFECT transfection reagents were purchased from Thermo Scientifics (Waltham, MA, USA). The Amplite™ Fluorimetric Catalase Assay Kit, Amplite™ Fluorimetric Glutathione Peroxidase Assay Kit, and Amplite™ Colorimetric Superoxide Dismutase Assay kit were purchased from ATT Bioquest (Sunnyvale, CA, USA).

### 4.2 Cell culture and neutral red survival assays

Human neuroblastoma SH-SY5Y cells were cultured in DME/F12 supplemented with 10% FBS and incubated in a 5% CO_2_ incubator at 37 °C. The passage number of cells was around 25 times starting from the 32nd generation. A neutral red uptake assay [71] with a slight modification [30] was adopted to measure cell viability. Briefly, after seeding (∼3 × 10^4^/cm^2^) for 2 days, cells were pretreated with or without SRT1720 for 1 h and then treated with or without PQ for another 24 h. Cells were loaded with neutral red (25 μg/mL), incubated at 37 °C for 2 h, washed once with 200 μL phosphate-buffered saline (PBS), and then added to 100 μL destaining solution (1% glacial acetic acid, 49% deionized H_2_O, and 50% ethanol [95%]). Each well's absorbance (540 nm) was measured using an enzyme-linked immunosorbent assay (ELISA) reader (Power Wave X; BioTek, VT, USA). After different treatments, cells growing on a 6-well plate were scraped and counted using a hemacytometer after trypan blue staining (0.3%).

### 4.3 Animal study

Male C57BL/6JNarl mice were purchased from the National Laboratory Animal Center (Taiwan). Mice were kept in a 12/12 h light/dark cycle and temperature (22 ± 2 °C) controlled room by following the principles and directives of the NIH Guide for the Care and Use of Laboratory Animals. The Institutional Animal Care and Use Committee reviewed and approved the experiments at the National Research Institute of Chinese Medicine (Approval No: 101-806-3). At 8 weeks, mice were injected intraperitoneally with or without PQ (15 mg/kg) twice a week for 4 weeks. Concomitantly, SRT1720 (0.1 and 1 mg kg) was also injected three times a week separately for 4 weeks. SRT1720 was injected 1 h before PQ treatment. In the fifth week after PQ treatments, the balance beam test was performed according to the previous study [7]. The beam consisted of a 1.5 m long strip of wood with a 5 × 20 mm^2^ cross-section. The number of hind leg slips of mice was recorded during their traversing through the beam. Mice were anesthetized by thiopental (50 mg/kg), perfused with saline and 4% paraformaldehyde, and decapitated for subsequent immunohistochemistry experiments after the beam walk test in the fifth week.

### 4.4 Caspase 3 activity assay

A Caspase 3 fluorometric assay kit (K105-100; Biovision Inc., Milpitas, CA, USA) was used to measure caspase 3 activity according to manufacturer instructions. In brief, cell extracts (50 μg/test) were reacted with DEVD-AFC (50 μM) for 1 h. Fluorescence (Ex/Em = 400/505 nm) was measured using a fluorescence microplate reader (M5, Molecular Devices, San Jose, CA, USA).

### 4.5 TUNEL assay

A TUNEL apoptosis assay kit (22849; AAT Bioquest, Sunnyvale, CA, USA) was used to detect apoptosis by following the instruction. In brief, cells growing on an 8-well chamber slide were stained with Tunnelyte™ for 30 min in an incubator. Cells were subjected to confocal analysis (LSM780; Carl Zeiss, Göttingen, Germany) after washing once with PBS and adding reaction buffer. Fluorescently labeled DNA strand breaks showing intense green fluorescent staining represented apoptotic cells.

### 4.6 Measurement of cellular reactive oxygen species (ROS)

ROS usually includes free-oxygen radicals (such as the superoxide anion radical and hydroxyl radical) and non-radical oxidants (including hydrogen peroxide and singlet oxygen). CellROX™ Orange Reagent is non-fluorescent in a reduced state and exhibits bright orange fluorescence (Ex/Em = 545/565 nm) upon ROS oxidation. Further, CellROX™ Green Reagent is a cell-permeant dye that is weakly fluorescent when in a reduced state and exhibits bright green photostable fluorescence upon oxidation by ROS and subsequent binding to DNA (Ex/Em = 485/520 nm). Thus, these dyes were used to measure cellular ROS. In brief, cells were loaded with 5 μM reagent for 30 min in an incubator, rinsed twice with PBS, and subjected to image acquisition with fixed exposure times with an inverted fluorescence microscope (Zeiss Axiovert 200 M; Carl Zeiss, Göttingen, Germany).

### 4.7 Measurement of mitochondrial membrane potential and ROS

After washing with Hank's Buffered Salt Solution, cells were stained with 100 nM TMRE (a marker for measuring mitochondrial membrane potential) for 15 min or 5 μM Mito-SOX (a marker of mitochondrial superoxide) for 10 min, where fluorescence intensity represents mitochondrial membrane potential and ROS. Cells were then subjected to image acquisition with fixed exposure times with an inverted fluorescence microscope (Zeiss Axiovert 200 M; Carl Zeiss, Göttingen, Germany). Imaging fluorescence intensities were quantified using ImageJ with the background subtracted. At least three frames were acquired for calculations for each treatment, and 15–30 cells per field were used for quantifications.

### 4.8 Gene silencing

According to standard protocols, the non-targeting control siRNA and SMART pools of siRNAs were used to silence the expressions of *NRF2*, *PGC-1α*, and *SIRT1* [39]. In brief, the mixtures were loaded to cells following the reaction of siRNAs and DharmaFECT Transfection Reagent in DME/F12 for 20 min and then incubated for 48 h. The volume proportion of 50 μM siRNA and DharmaFECT was 1:4, and the working concentration of siRNA was 50 nM.

### 4.9 Western blot analysis

Western blot analysis was performed as described previously [39]. In brief, equal amounts of cell lysates (∼20 μg/well) derived from the filtered assay were separated by SDS-polyacrylamide gel electrophoresis (SDS-PAGE) and then electroblotted onto Immobilon polyvinylidene difluoride (PVDF) membranes (Millipore). Membranes were blocked with 5% skim milk in TBST (100 mM Tris-HC1 and 150 mM NaC1 at pH 7.4 containing 0.05% Tween 20) for 1 h at room temperature and then incubated with the first antibody (1:1000–2000) at 4 °C overnight. After three washes with TBST, the blot was incubated with a second antibody (1:5000–10000) conjugated to horseradish peroxidase for 1 h, processed for visualization using an enhanced chemiluminescence system (Pierce, Rockford, IL, USA), and exposed to Fuji medical X-ray film (Super RX-N, FUJIFILM Corporation, Tokyo, Japan) to obtain fluorographic images.

### 4.10 Dot blot analysis

As described in the protocol of Western blot analysis, equal protein concentrations (10 μg/well) in each group were filtered through a nitrocellulose membrane using the Bio-Dot Microfiltration Apparatus (Bio-Rad, Hercules, CA, USA). Each well was washed twice during suction with 200 μL TBST (100 mM Tris-HCl, 150 mM NaCl, and 0.1% Tween 20; pH 7.4). The transferred blot was blocked in TBST containing 5% skim milk for 1 h at room temperature and then incubated with the antibody in 3% bovine serum albumin (BSA) with 0.02% NaN_3_ at 4 °C overnight. The following method was performed according to the protocol of Western blot analysis.

### 4.11 Transient transfection and image detection

Lipofectamine 2000 was used as a vehicle to transfer plasmids into cells according to the manufacturer's protocol. Briefly, 1 μg of DNA combined with 1 μl of Lipofectamine 2000 was applied to each well of 24-well plates (approximately 1.2 × 10^5^ cells/cm^2^). After transfection for 24 h, cells were treated with reagents for another 24 h. Cells were fixed, mounted, and observed with a confocal microscope.

### 4.12 Aggresome assay

The PROTEOSTAT® Aggresome Detection Kit (ENZ-51035-0025; Enzo Life Sciences, Farmingdale, NY, USA) was used to monitor the presence of aggresomes. In brief, after fixation and permeabilization, cells were loaded with PROTEOSTAT® aggresome red dye and Hoechst stain, and then subjected to image acquisition with confocal microscopy.

### 4.13 Proteasome activity assay

The Proteasome-Glo™ Chymotrypsin-Like Assay Kit (G8622; Promega, Madison, WI, USA) was used to measure proteasome activity. Cells were collected and homogenized; equal concentrations of proteins were added to Proteasome-Glo™ buffer, Suc-LLVY-Glo™ substrate, and luciferin detection reagent to separate groups; and luminescence was detected with a luminometer (GM2000; Promega).

### 4.14 Antioxidative enzyme activity assay

The Amplite™ Fluorimetric Catalase Assay kit was used to measure catalase activity. In brief, an equal concentration and volume of a sample from each group were added to the H_2_O_2_ assay buffer and catalase assay buffer sequentially and then incubated at room temperature for 15 min. Fluorescence was measured at Ex/Em = 540/590 nm. The Amplite™ Fluorimetric Glutathione Peroxidase Assay kit was used to measure GPX activity. In brief, an equal concentration and volume of a sample from each group were reacted with GPX working solution for 30 min, a Quest™ Flour NADP probe for 10 min, and an enhancer solution for 30 min. Fluorescence was then measured at Ex/Em = 420/480 nm. An Amplite™ Colorimetric Superoxide Dismutase Assay kit was used to measure SOD activity. In brief, an equal concentration and volume of the sample in each group was added to SOD working solution I/II and incubated at room temperature for 30 min. Absorbance was measured at 560 nm using an ELISA reader.

### 4.15 Immunocytochemistry (ICC)

Cells were spread and grown on sterile glass coverslips. After various treatments, cells were fixed for 10 min with 4% paraformaldehyde at room temperature. Cells were rinsed three times with PBS after fixation. Further, cells were permeabilized using 0.5% Triton-100 in PBS for 15 min. A blocking agent composed of 10% goat serum and 0.3% Triton-100 in PBS was applied at room temperature for 90 min to reduce nonspecific antibody activity. After three washes with PBS, cells were labeled with antibodies (1:200), dissolved in 1% BSA and 0.3% Triton-100 in PBS, and incubated at 4 °C overnight. After labeling, cells were washed three times with PBS at room temperature. Cells with IgG fluorescein-conjugated secondary antibodies (1:200) were incubated in 1% BSA/PBS at room temperature for 90 min and then counterstained with 4′,6-diamidino-2-phenylindole (DAPI; 1 μg/ml) at room temperature for 10 min if required. Cells were washed 3 times with PBS and then mounted on microscope slides with Aqua Poly-Mount (Polysciences, Warrington, PA, USA).

### 4.16 Immunohistochemistry (IHC)

Brains from decapitated mice were immersed in 30% sucrose and 4% paraformaldehyde/PBS. After dissection (20 μm/section), brain slices were permeabilized and blocked with 10% GS and 0.1% BSA in IHC buffer (0.1% Triton-100, 0.05% Tween-20, and 0.05% NaN_3_ in PBS) at room temperature for 1 h. After three washes with IHC buffer, brain slices were labeled with antibodies (1:200) dissolved in 1% GS and 0.05% BSA/IHC buffer, and held at 4 °C overnight. After three washes with IHC buffer, slices were incubated with IgG fluorescein-conjugated secondary antibodies (1:200) in the presence of DAPI (1 μg/ml) in IHC buffer at room temperature for 60 min. Brain slices were washed three times with IHC buffer and then mounted on microscope slides with Aqua-Poly/Mount.

### 4.17 Statistics

Statistical analyses were performed with SigmaPlot Version 14.5. Data are expressed as mean ± standard deviation (SD). Differences among groups were assessed by nonparametric Kruskal-Wallis analysis of variance (ANOVA) on ranks or one-/two-way ANOVA. Post hoc comparisons were calculated using the Student-Newman-Keuls test, and the results were considered significant at *p* < 0.05.

## Author contributions

T.H.H, C.N.Y., and P.Y.L. performed most of the experiments; C.C.C. and C.L.H designed and performed part of the experiments; J.J.C., I.J.L., C.T.C., and Y.C.Y. supervised the project; J.J.C. and N.K.H. proposed the project and wrote the manuscript. All authors read and approved the final manuscript. We thank Mr. B.C. Pruitt, Jr. for English-language editing of the manuscript.

## Fundings

This work was supported by the National Research Institute of Chinese Medicine (MOHW110-NRICM-B-325-112104), 10.13039/501100009974Cardinal Tien Hospital (CTH108A-2A06; CTH109A-2201), and the 10.13039/501100001868National Science Council, ROC (10.13039/501100004663MOST 109-2320-B-077-005-MY3), Ministry of Health and Welfare (MOHW111-NRICM-M-315-134001).

## Declaration of competing interest

The authors assert no conflict of interest in this investigation.

## Data Availability

Data will be made available on request.
