# Supplementary material for: SRT1720 as an SIRT1 activator for alleviating paraquat-induced models of Parkinson's disease
Source: Redox Biol. 2022 Nov 11;58:102534. doi: 10.1016/j.redox.2022.102534 (PMC9663539; doi:10.1016/j.redox.2022.102534)
Supplement: Multimedia component 1 [file mmc1.docx]

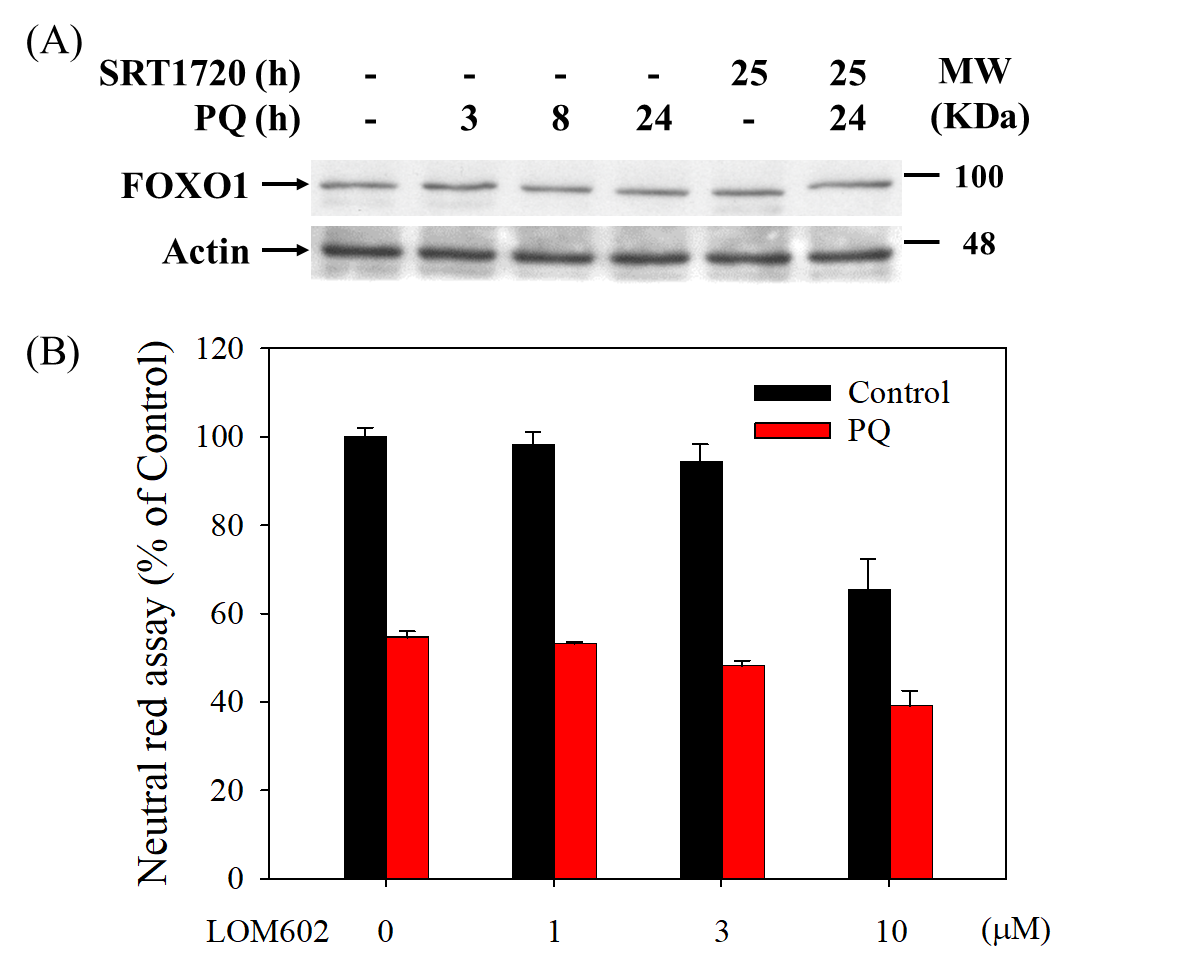


**Supplementary Fig. 1. Effects of SRT1720 on FOXO1 expressions and LOM612 (a relocator of FOXO1) during PQ treatment.**

**Supplementary Fig. 2. AGK2, an SIRT2 inhibitor, failed to block PQ-induced cell death in human SH-SY5Y cells.**

**Supplementary Fig. 3. Effect of SRT1720 on PGC-1α promoter activity during different treating interval.**

**
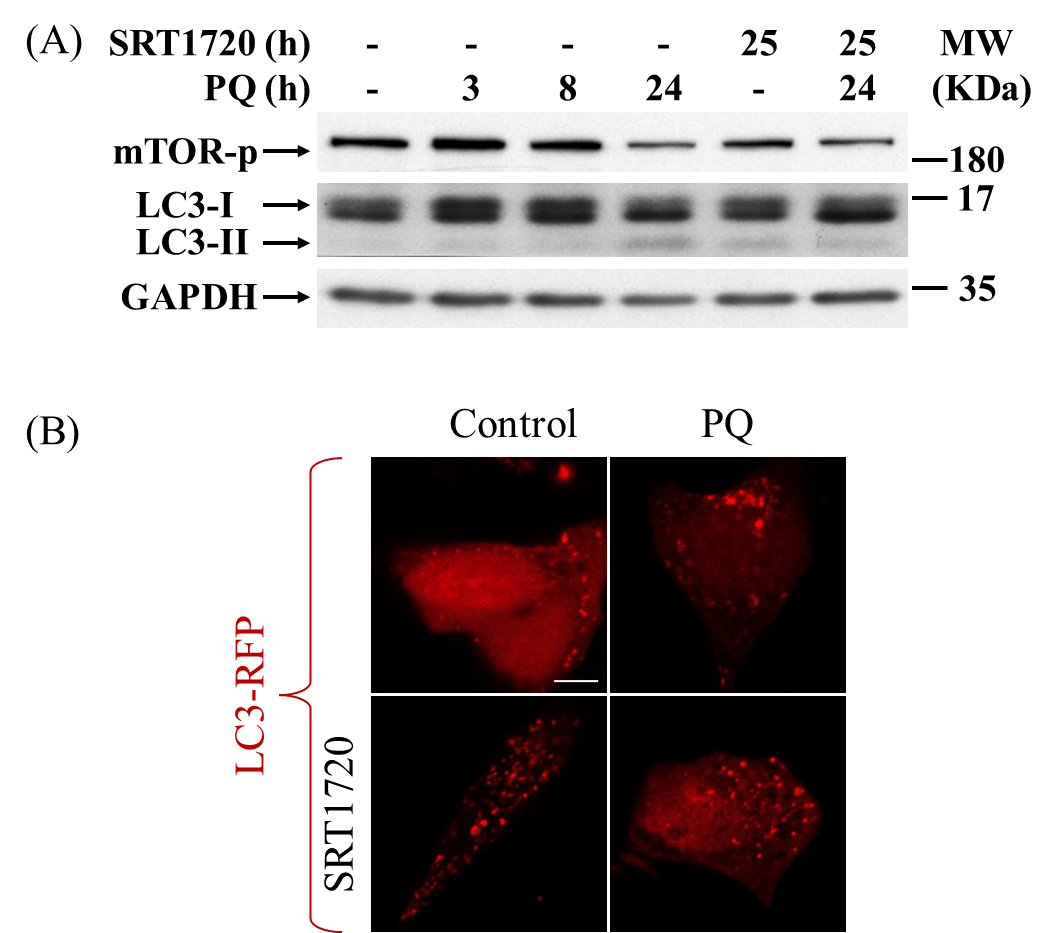
**

**Supplementary Fig. 4. Effect of SRT1720 and PQ on autophagy and its related markers.**

**
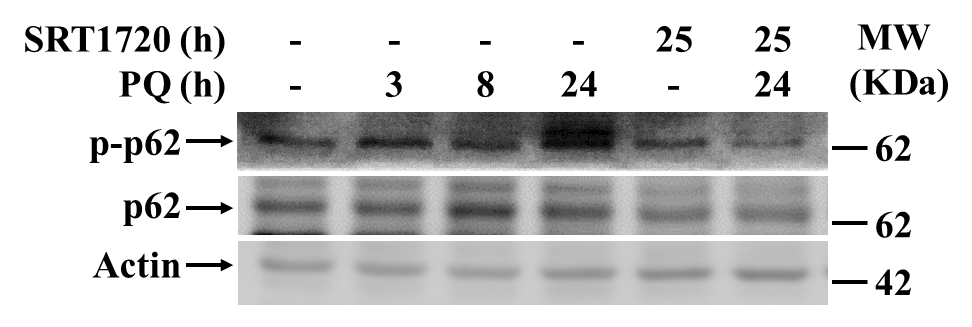
**

**Supplementary Fig. 5. Effect of SRT1720 and PQ on the expressions of phosphorylated and non-phosphorylated forms of p62.**


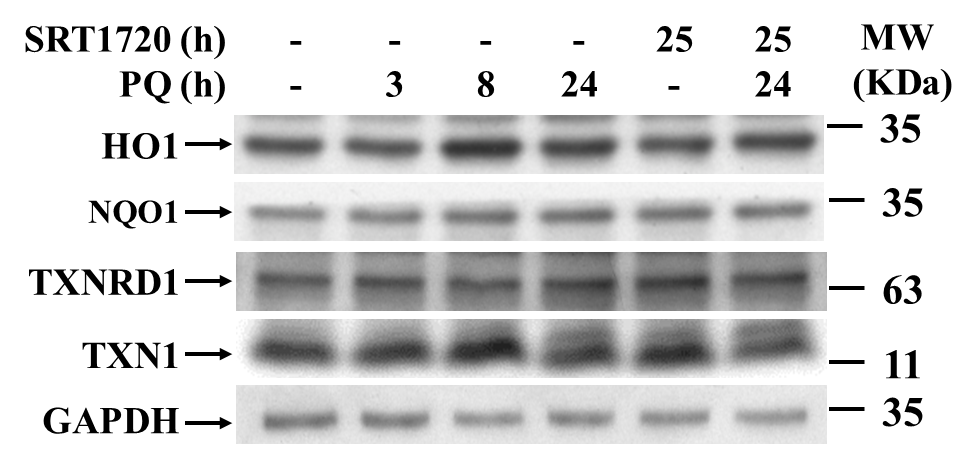


**Supplementary Fig. 6. Effect of SRT1720 and PQ on the expressions of HO1, BQO1, TXNRD1, and TXN1.**

**
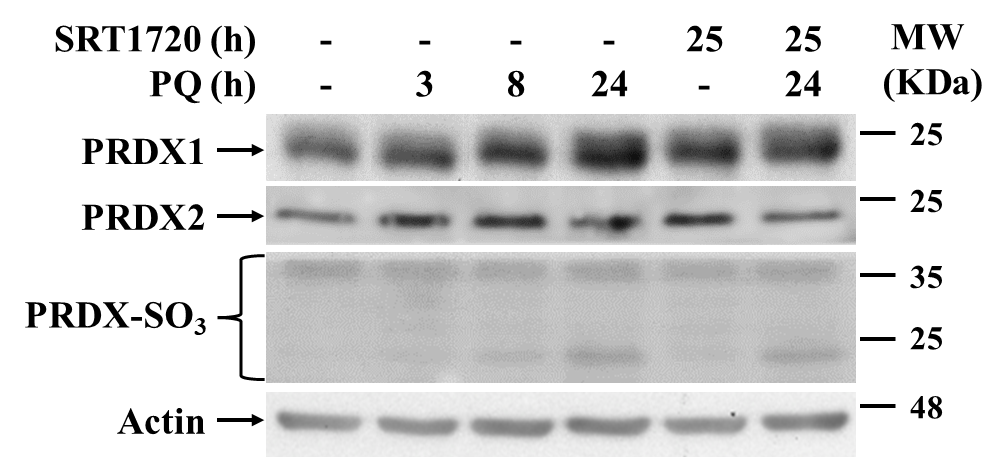
**

**Supplementary Fig. 7. Effect of SRT1720 and PQ on the expressions of PRDX1, PRDX2, and PRDX-SO_3_.**
